# O051. Chronic migraine and onabotulinumtoxinA: a prospective study on patients treated at the Headache Centre of the Padua University and analysis of possible predictors of responsivity

**DOI:** 10.1186/1129-2377-16-S1-A98

**Published:** 2015-09-28

**Authors:** Caterina Disco, Matteo Bellamio, Matteo Fuccaro, Martina Bruno, Alberto Terrin, Federico Mainardi, Giorgio Zanchin, Ferdinando Maggioni

**Affiliations:** Department of Neurosciences, Headache Centre, University of Padua, Padua, Italy; Department of Neurology, Headache Centre, Hospital SS Giovanni and Paolo, Venice, Italy

## Background

Real clinical setting data about onabotulinumtoxinA treatment on chronic migraine are poor, especially in patients with medication-overuse headache (MOH) and ≥ 65 years old, as well as data on predictors of responsivity. W present results on chronic migraine patients treated at the Padua Headache Centre from April 2014 to March 2015.

## Materials and methods

By compiling a headache diary, efficacy parameters (mean reduction of headache days and hours) were evaluated at 90 days after the first cycle. We analyzed also: 30% and 50% response rates and the percent of first-time responders to II cycle; association with headache related symptoms and comorbidities (depression/anxiety disorders, hypertension, sleep disturbances, caffeine intake, BMI >30).

## Results

Forty patients were evaluated (35 F, 5 M; mean age, 53±12.8) of which 37/40 (93%) with MOH. At 90 days after the first cycle headache diary documented a significant mean reduction of headache days (56.2 vs 69.2, p < 0.005), of the total hours of headache (455.4 vs 601.6, p <0.005), of the hours of moderate pain (147.8 vs 263.5, p < 0.005) and severe pain (102.5 vs 131.2, p < 0.05), of the consumption of triptans (30.7 vs 46.5, p < 0.001) and associations (15.4 vs 22.7, p <0.05). The 8 patients ≥65 years old did not present a significant reduction of efficacy parameters vs younger patients. 50% and 30% response rate was respectively 22.5% and 38% for at least one efficacy parameter, 15% and 25% for headache days, of 20% and 35% for hours, 12.5 and 23% for both parameters. Percent of “first-time 50% responders” was 15.8% and 10.8% respectively for headache days and hours; percent of “first-time 30% responders” was 26.3% and 15.8%. Cluster analysis showed a higher severe headache share and a lower share of mild headache in responsive patients vs non responsive: 146.7 (26.3%) vs 119.1 (17.6%) severe pain hours, 162.2 (29.1%) vs 287.1 (41.1%) mild pain hours. ANOVA analysis did not show significant association between responsivity and headache symptoms or related comorbidities, except for a lower response trend of depression/anxiety at limit of significance (p = 0.07).

## Conclusions

OnabotulinumtoxinA treatment appears useful also in a clinical setting with high presence of MOH. Responsive patients are <65 years old and have a higher frequency of severe headache and a lower share of mild headache. Depression/anxiety disorders are associated also to a lower responsiveness trend at limit of significance.

Written informed consent to publication was obtained from the patient(s).

## Conflict of interest

The principal author declares that there is no conflict of interest.

